# Integrating Surface Plasmon Resonance and Docking Analysis for Mechanistic Insights of Tryptase Inhibitors

**DOI:** 10.3390/molecules30061338

**Published:** 2025-03-17

**Authors:** Alessia Porta, Candida Manelfi, Carmine Talarico, Andrea Rosario Beccari, Margherita Brindisi, Vincenzo Summa, Daniela Iaconis, Marco Gobbi, Marten Beeg

**Affiliations:** 1 Istituto di Ricerche Farmacologiche Mario Negri IRCCS, Via Mario Negri, 2, 20156 Milano, Italy; alessia.porta@marionegri.it (A.P.); marten.beeg@marionegri.it (M.B.); 2 EXSCALATE-Dompé Farmaceutici SpA, via Tommaso De Amicis 95, 80131 Napoli, Italy; candida.manelfi@dompe.com (C.M.); carmine.talarico@dompe.com (C.T.); andrea.beccari@dompe.com (A.R.B.); 3 Department of Pharmacy, School of Medicine and Surgery, University of Naples Federico II, Via D. Montesano 49, 80131 Naples, Italy; margherita.brindisi@unina.it (M.B.); vincenzo.summa@unina.it (V.S.)

**Keywords:** tryptase inhibitors, molecular interactions, molecular modeling, surface plasmon resonance, in silico docking, X-ray crystal structures

## Abstract

Tryptase is a tetrameric serine protease and a key component of mast cell granules. Here, we explored an integrated approach to characterize tryptase ligands, combining novel experimental binding studies using Surface Plasmon Resonance, with in silico analysis through the Exscalate platform. For this, we focused on three inhibitors previously reported in the literature, including a bivalent inhibitor and its corresponding monovalent compound. All three ligands showed concentration-dependent binding to immobilized human tryptase with the bivalent inhibitor showing the highest affinity. Furthermore, Rmax values were similar, indicating that the compounds occupy all four binding pockets of the tryptase tetramer. This hypothesis was supported by in silico computational analysis that revealed the binding mode of the monovalent ligand, one in each monomer pocket, compared with crystal structure of the bivalent one, which simultaneously occupies two binding pockets. Additionally, we solved the 2.06 Å X-ray crystal structures of human Tryptase beta-2 (hTPSB2), in both its apo form and in complex with compound #**1**, experimentally confirming the binding mode and the key molecular interactions predicted by docking studies for this compound. This integrated approach offers a robust framework for elucidating both the strength and mode of interaction of potential tryptase inhibitors.

## 1. Introduction

Mast cells are immune cells derived from myeloid progenitors that play a crucial role in inflammatory responses, neuroimmune regulation, and tissue remodeling. These cells contain granules rich in bioactive molecules, including histamine, heparin, and proteases, which are released upon activation [1]. Among these proteases, tryptase, a tetrameric serine protease, is one of the most abundant and functionally significant enzymes in mast cell granules [2,3]. Tryptase is implicated in extracellular matrix (ECM) remodeling and inflammatory diseases, including asthma, chronic pain, fibrosis, and cancer progression [4,5]. Due to its central role in mast cell-mediated responses, tryptase inhibition has emerged as a promising therapeutic strategy for conditions linked to excessive mast cell activation [6,7].

Despite the strong therapeutic interest in tryptase inhibitors, a detailed understanding of their binding stoichiometry and mechanism of action remains incomplete. While many inhibitors have been evaluated based on potency, their exact mode of interaction with the tryptase tetramer is not well characterized at the molecular level. In particular, bivalent inhibitors—which engage multiple active sites simultaneously—have shown increased potency compared to monovalent compounds, yet the molecular basis of this enhanced affinity is not fully understood.

**Figure 1:**
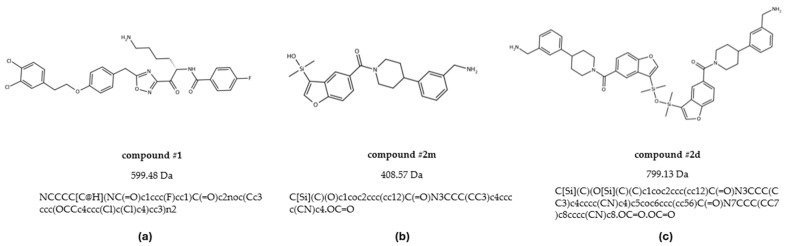
Structure, molecular weight and SMILE of compounds #**1** (**a**), #**2m** (**b**), and #**2d** (**c**).

To address this gap, we employed an integrated approach that combines computational and experimental methodologies to investigate both binding affinity and stoichiometry of tryptase inhibitors, with the additional aim to cross-validate these methodologies for more reliable screening of novel compounds. For this, in the present study we resynthesized and examined three tryptase inhibitors reported in the literature (Figure 1) [8,9].

To experimentally determine the binding properties, we exploited Surface Plasmon Resonance (SPR) technology, which allows us to measure the association and dissociation rate constants and, as a consequence, to provide information on the binding mode (e.g., on the presence of avidity effects, possibly exhibited by bivalent inhibitors) and on the binding stoichiometry [10,11]. It was already described that bivalent inhibitors usually enhance binding affinity compared to the monovalent ones by exploiting the ability to occupy two binding sites on a target molecule simultaneously through a cooperative effect [12]. Gathering this information during drug discovery helps to design more effective drugs, and the SPR approach provides these data quickly and cost effectively. Molecular docking simulations with the Exscalate platform were then applied to obtain the molecular details of the interaction of these inhibitors within the tryptase tetramer and to support the experimental findings. In order to further validate our findings, the X-ray crystal structure of one inhibitor–tryptase complex was solved, confirming the data.

By integrating in silico predictions with biophysical and structural validation, this study offers new insights into the molecular mechanisms of tryptase inhibition. The findings not only enhance our understanding of ligand–tryptase interactions but also support the rational design of more potent and selective tryptase inhibitors for therapeutic applications.

## 2. Results

### 2.1. Surface Plasmon Resonance Experimental Validation

For the present studies, aiming at investigating the binding properties of small molecules flowing over human tryptase immobilized on the sensor chip, we used a highly sensitive SPR instrument, and a chip allowing high immobilization levels. The experimental conditions were optimized, as described in the Methods section, to have adequate and reproducible binding signals. 

Each of the three investigated compounds (Figure 1: #**1**, #**2m**, #**2d**) was tested in at least three independent SPR experimental sessions and injected at different concentrations.

**Figure 2:**
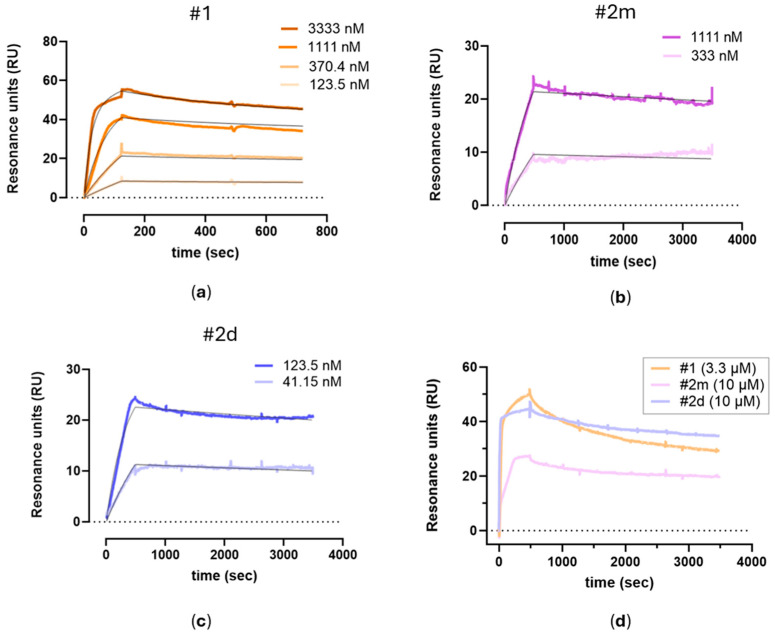
Representative SPR sensorgrams (i.e., the time course of SPR binding signals, in Resonance Units, RU) were obtained by injecting different concentrations of #**1** (**a**), #**2m** (**b**), and #**2d** (**c**) over human tryptase immobilized on the sensor chip. For each compound, the sensorgrams were fitted globally using a two-site binding model, with the resulting fitting curves represented by black lines. (**d**) Overlay of SPR sensorgrams at saturating concentrations: 3.3 µM for compound #**1** and 10 µM for compounds #**2m** and #**2d**.

Representative sensorgrams are shown in Figure 2a–c to highlight the concentration-dependent binding and the good interpolation of the data with a global fitting (i.e., considering both the association and the dissociation phases at all the compound’s concentrations). For these fitting, we needed to use an equation including two binding sites, suggesting the presence of a specific high-affinity interaction occurring at lower drug concentrations, while at higher concentrations, a lower affinity interaction also appears. Figure 2d reports representative sensorgrams obtained with a saturating concentration of the compounds for a more reliable determination of the Rmax values.

The affinity constants of the high-affinity component obtained in each independent experiment, together with the mean values, are reported in Table 1 to allow for appreciation of the inter-assay variability. The high-affinity site was chosen as it accounts for the majority of interactions and is therefore possibly more relevant to the overall binding mechanism. The compound with the highest affinity (K_D_ = 2.1 nM) was the bivalent inhibitor #**2d**, whereas the one with the lowest affinity (K_D_ = 81 nM) was the corresponding monovalent compound #**2m**, while compound #**1** showed an intermediate affinity (K_D_ = 12.3 nM). Considering the two monomers, the higher affinity of compound #**1** in comparison with compound #**2m** is mainly due to a 17-fold faster association, partially counteracted by a 3-fold faster dissociation; the comparison of monomeric compound #**2m** with the corresponding dimeric compound #**2d** shows that the higher affinity of the latter is also mainly due to a faster association (38-fold) while dissociation constants are similar.

As reported in Table 1, the Rmax value of the monomer #**2m** (26.3 ± 3.5 RU, MW409) was similar to the Rmax value of the dimer #**2d** (32.8 ± 5.6 RU, MW 800). A similar Rmax can be also calculated for compound #**1**, taking into account that its MW (600 RU) is 1.5-fold that of compound #**2m**, resulting in a normalized Rmax of 29 ± 3.2 RU. These data indicate that all four binding pockets of the tryptase tetramer are occupied but with different stoichiometric ratios. Monovalent compounds (#**1** and #**2m**) follow a 4:1 stoichiometry, with four molecules binding per enzyme, whereas the bivalent compound (#**2d**) follows a 2:1 stoichiometry, binding two molecules per enzyme. This suggests that monovalent inhibitors engage each active site independently, while the bivalent inhibitor spans two adjacent sites.

### 2.2. Molecular Docking Supports Affinity and Binding Stoichiometry of Tryptase Inhibitors

The SPR studies provided insights into the binding kinetics, affinities, and binding stoichiometry of compounds #**1**, #**2m**, and #**2d**. To further characterize these interactions, docking studies were performed to explore their binding modes.

While the binding mode of bivalent inhibitor #**2d** has been previously characterized (PDB: 4MPW) [8], the exact interaction patterns of monovalent inhibitors, including compounds #**1** and #**2m**, required further investigation.

In order to explore and validate these binding modes through molecular docking, we generated X-ray crystal structures of human tryptase beta-2 (hTPSB2) in its apo form.

**Figure 3:**
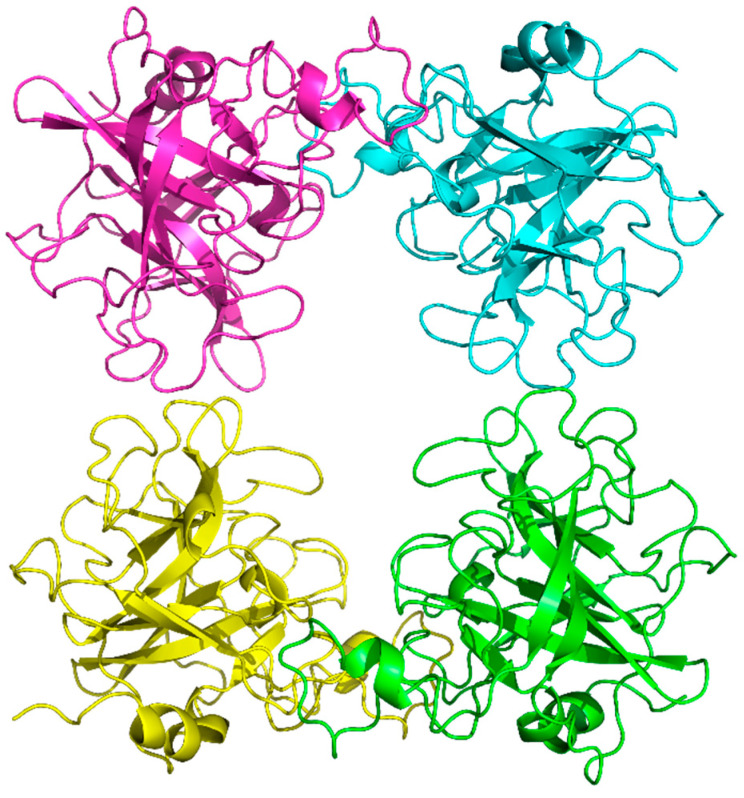
Overall structure of hTPSB2 tetramer shown as ribbon diagram.

The structure was solved at a resolution of 2.06 Å and displayed the typical trypsin-like serine protease fold. It forms a homotetramer, with the catalytic sites facing toward the central channel (Figure 3).

**Figure 4:**
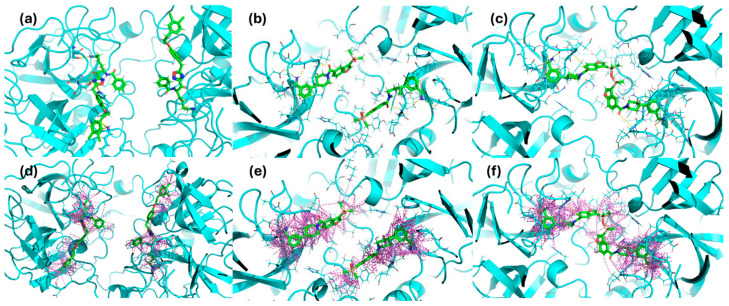
Docking-predicted binding modes of compound #**1** (**a**,**d**), compound #**2m** (**b**,**e**), and compound #**2d** (**c**,**f**), within the tryptase active site respectively showing H-bond networks (yellow dashed lines panel (**a**–**c**)) and hydrophobic contacts (purple dashed lines panel (**d**–**f**)).

The docking studies presented in Figure 4 revealed distinct binding interactions among the inhibitors, with compound #**1** exhibiting a higher number of polar interactions, five hydrogen bonds with key catalytic site residues instead of the three ones estabilished by compound #**2m**, and a more extended network of hydrophobic contacts, reaching a new larger portion of the protein, compared to compound #**2m**. Also, comparison between compound #**2m** and compound #**2d** shows a more efficient binding mode with additional hydrophobic interactions involving the central disiloxane linker of the bivalent compound #**2d**, in agreement with experimental results showing an increase in the binding affinity.

**Figure 5:**
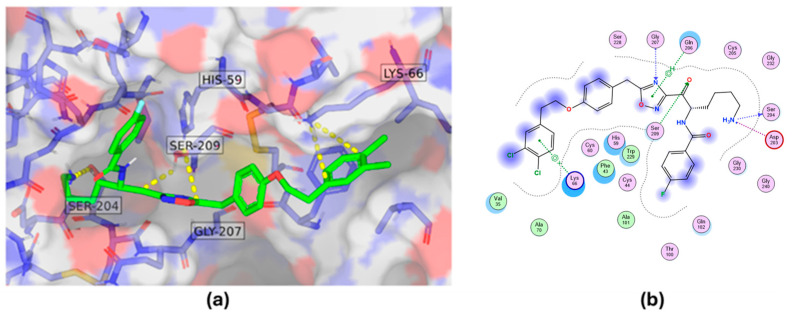
Docking binding mode showing interactions of compound #**1** with key residues of tryptase. Three-dimensional binding interaction representation (**a**) and two-dimensional binding interaction map (**b**).

In-depth analysis of compound #**1** revealed that it engages key catalytic site residues via hydrogen bonds to the side chain atoms of Ser209, His59, and Lys66, as well as hydrogen bonds to the main chain atoms of Gly207 and Ser204 (Figure 5).

In particular, the H-bond network between the ketone group of the ligand and the hydroxyl group of the residue Ser209 suggests a potential covalent binding mode for compound #**1** [13]. In order to verify this hypothesis, we applied a covalent docking procedure confirming our assumption: the covalent mechanism is favored and the binding mode is very similar to the previous one described.

**Figure 6:**
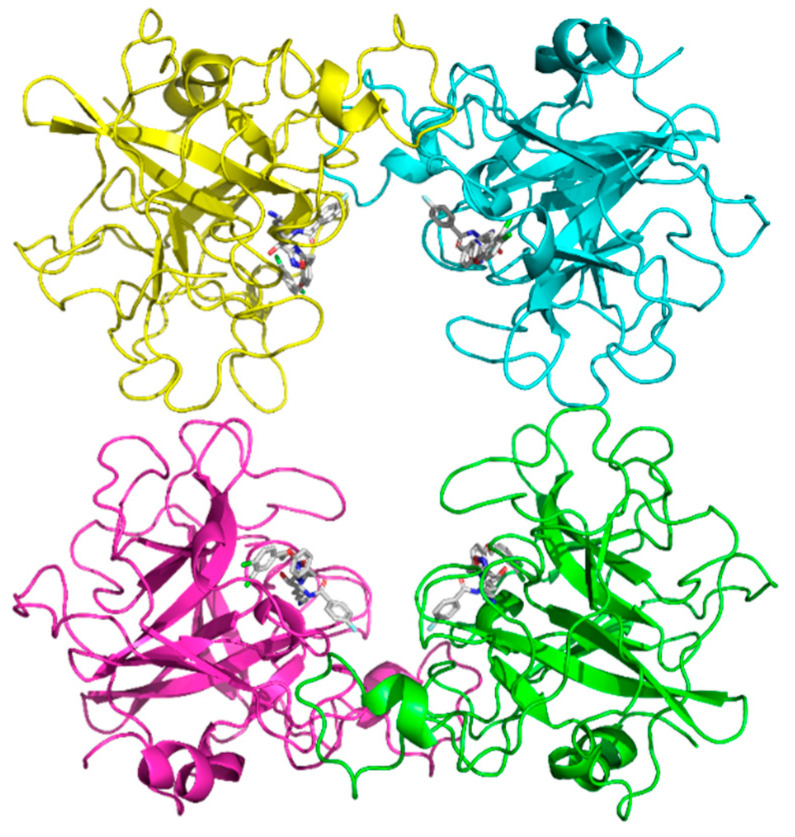
Overall structure of hTPSB2, containing compound #**1**, shown as ribbon diagram.

To experimentally confirm the effective mechanism of action of compound #**1**, we have solved the crystal structure of its complex with tryptase (Figure 6).

**Figure 7:**
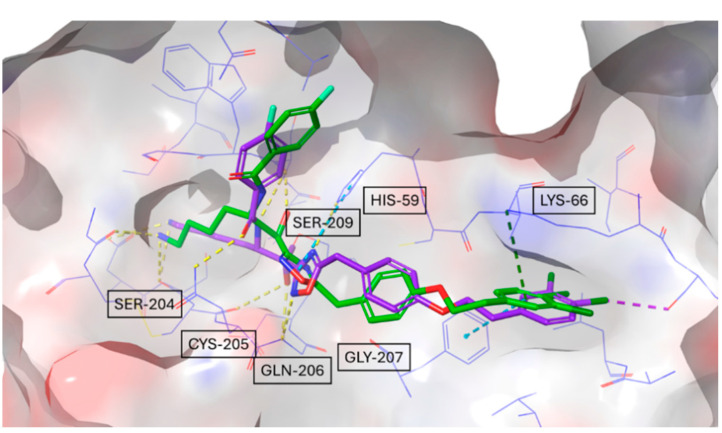
Overlay of the docking-predicted binding mode of compound #**1** (green) with its experimentally determined crystal structure (violet).

Based on a distance of less than 3.5 Å of the donor and acceptor atoms, we identify nine specific hydrogen bonds of the ligand compound #**1**, namely to the main chain atoms of Ser204, Ser228, Cys205, Gly207, and Ser209, as well as the side chain atoms of Ser204, Gln206, and His59. According to the above distance criteria, we also suggest the presence of additional hydrophilic interactions with the main chain atoms of Trp229, Ile241, Gly232, and Arg238. The crystal structure of the complex confirms the binding mode hypothesized by docking studies, retrieving almost all the key interactions (Figure 7).

**Figure 8:**
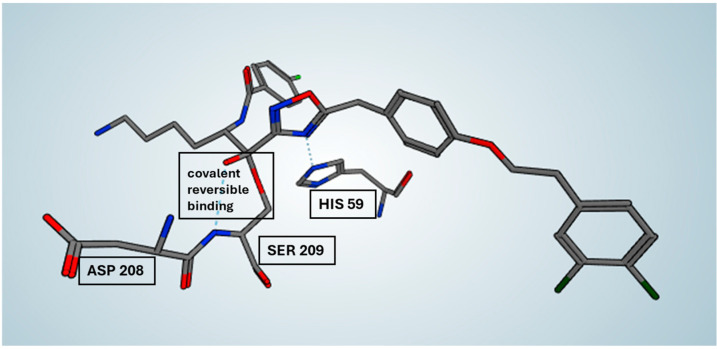
Focus on the reversible covalent binding mode of compound #**1** (violet) in the X-ray complex structure with tryptase.

The experimental electron density clearly shows that the compound #**1** is covalently bound with C12 of the electrofilic carbonyl group with the hydroxyl group of the active site serine residue, Ser209, forming a reversible hemiketal (hemiacetal), in agreement with the SPR-derived Kd values. In particular, it represents an example of covalent-reversible inhibition of serine protease (Figure 8).

## 3. Discussion

The integrated SPR and computational approaches described here offer a robust framework for elucidating both the strength and mode of interaction of potential tryptase inhibitors.

To our knowledge, this is the first study determining the binding constants of tryptase inhibitors, whose activity is usually measured by functional assays. For this aim, we developed a novel SPR-based assay in which the inhibitors flow over human tryptase immobilized on a sensor chip. The assay proved to be sensitive and reproducible, with a relatively low inter-assay variability.

In particular, SPR data provided clear evidence for binding stoichiometry—distinguishing monovalent from bivalent binding modes—even in the absence of crystal structures. This capability is crucial in guiding rational drug design, as seen by the dramatic increase in binding affinity when the monomeric ligand (#**2m**) is converted into its dimeric counterpart (#**2d**), yielding a 40-fold improvement. These results correlate well with functional assays (IC_50_ = 2.51 nM for #**2d** vs. 446 nM for #**2m**) [8,9] and illustrate how detailed kinetic and stoichiometric insights can inform more targeted lead optimization.

**Figure 9:**
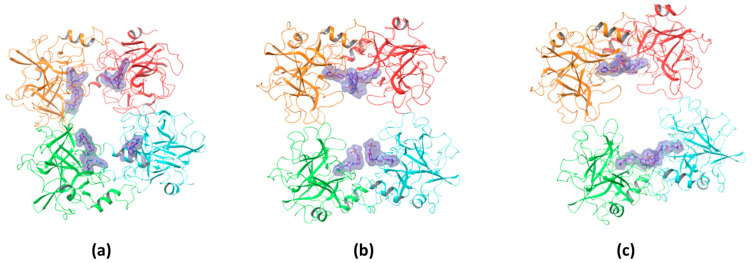
Binding stoichiometry of compounds #**1** (4:1) (**a**), #**2m** (4:1) (**b**), and #**2d** (2:1) (**c**) within the tryptase tetramer.

The binding stoichiometry findings were further supported by structural and computational data. The X-ray crystal structure of compound #**1** confirmed a 4:1 stoichiometry, showing that four molecules bind per tryptase tetramer, each occupying an independent active site (Figure 9a). Similarly, the previously resolved complex of the bivalent inhibitor (PDB: 4MPW) validated a 2:1 stoichiometry, where compound #**2d** spans two adjacent monomers, bridging their active sites (Figure 9c). These findings illustrate that monovalent inhibitors bind independently to each of the four active sites, while bivalent inhibitors engage two adjacent monomers, reducing the overall occupancy ratio.

While direct structural confirmation for compound #**2m** is not yet available, docking results indicate that its binding mode closely resembles that of compound #**1**, supporting the experimentally determined 4:1 stoichiometry (Figure 9b). Additional experimental evidence further supports this hypothesis; in particular, the crystal structure of the tryptase tetramer bound to a monovalent inhibitor (PDB: 2ZEB) shows a binding mode similar to that suggested by docking for compound #**2m** [14]. Together, these findings strongly suggest that compound #**2m** also binds to separate active sites within the tetramer, reinforcing the role of SPR and docking in predicting binding modes even in the absence of crystallographic data.

Furthermore, the docking simulations revealed distinct hydrogen bonding and polar interaction patterns among the inhibitors, providing structural insights into their experimentally observed binding affinities. Compound #**1** exhibited the highest number of hydrogen bonds and polar contacts, forming multiple stabilizing interactions within the tryptase active site. In contrast, compound #**2m** engaged in fewer hydrogen bonds and lacked additional polar interactions, which may contribute to its higher K_D_ value observed in SPR experiments.

Compound #**2d**, as a bivalent inhibitor, displayed approximately twice the number of hydrogen bonds and polar interactions per molecule compared to compound #**2m**. By spanning two adjacent active sites, it effectively doubled the interaction network, likely resulting in its significantly stronger binding affinity. These findings illustrate how differences in hydrogen bonding and polar contacts correlate with binding strength, reinforcing the role of docking studies in explaining experimentally determined affinities. Building on this, we examined whether bivalent inhibitors induce structural rearrangements in tryptase, as such conformational changes could contribute to the observed kinetic complexity. A comparison of the crystal structures of tryptase complexed with compound #**1** and the previously resolved structure of tryptase bound to compound #**2d** revealed an RMSD value of 0.53 Å, indicating that no significant conformational changes occurred within the tryptase tetramer upon binding either monovalent or bivalent inhibitors. This finding suggests that the multi-step binding behavior observed in SPR arises primarily from the nature of the non-covalent interactions rather than from allosteric effects. However, considering the complex sensorgram fitting required to describe the binding behavior, it remains possible that factors such as heterogeneous immobilization of tryptase or differential exposure of active sites contribute to the observed kinetics. The docking studies not only provided insights into hydrogen bonding and polar interactions but also suggested that compound #**1** could form a reversible covalent bond with the catalytic Ser209 residue. This hypothesis was supported by the proximity of these functional groups in the binding pose, and subsequent covalent docking confirmed a likely reversible covalent interaction mode. This prediction was also confirmed by crystallographic analysis. SPR analysis of the compound #**1** revealed a binding mechanism that could not be fully described by a simple 1:1 interaction model, instead requiring a more complex kinetic fitting approach (two state model). Reversible covalent inhibitors often follow a two-step binding process: an initial non-covalent association, followed by a slower covalent bond formation. This two-step mechanism can generate complex binding kinetics, resembling a two-site binding model even when only a single binding site is involved. Additionally, the data showed a notable dissociation rate, suggesting a dynamic yet stable engagement with the active site. A similar observation was reported by Akçay et al., who studied reversible covalent inhibitors targeting a lysine residue [15]. Their SPR data also required a complex binding model, rather than a simple 1:1 interaction, and showed notable dissociation rates, reinforcing the idea that reversible covalent inhibitors often follow multi-step binding mechanisms. The integration of SPR, docking, and crystallography in this study highlights the power of computational–experimental workflows in drug discovery. While classical docking was used to characterize ligand binding, these approaches could be further enhanced by Artificial Intelligence-Driven Drug Discovery (AIDD) methods. AI and machine learning models, when trained on quantitative kinetic data (SPR), structural insights (X-ray crystallography), and ligand-binding predictions (docking), may have the potential to systematically identify and optimize novel ligands with greater accuracy. Incorporating AI-based strategies could refine virtual screening, improve binding affinity predictions, and streamline structure-based drug design. These advancements represent a natural extension of the computational methods employed in this study, potentially accelerating the identification of improved tryptase inhibitors.

## 4. Materials and Methods

### 4.1. Compounds

The protein used for crystallization was recombinant human tryptase beta-2 (hTPSB2), purchased from Promega (Madison, WI, USA; Cat. No. G5631). hTPSB2 was selected for this study based on preliminary screening data indicating its upregulation in a specific tissue of interest, supporting its relevance as a therapeutic target. The inhibitors studied in this work were compound #**1**, compound #**2m**, and compound #**2d**. These compounds were synthesized, purified, and characterized following the literature procedure with slight modifications [8,9].

### 4.2. Surface Plasmon Resonance Assays

All the SPR studies were carried out by using the very sensitive Sierra SPR-32pro from Bruker (Billerica, MA, USA). Human tryptase B2 (Promega, G5631, Madison, WI, USA) was immobilized via amine coupling on a High-Capacity Amine chip (Bruker, Billerica, MA, USA). After surface activation with EDC (400 mM) and NHS (50 Mm), the protein was injected at 30 µg/mL in acetate buffer pH 5.5 (10 min at 5 µL/min). The residual active sites were then inactivated with 1 M ethanolamine (6 min at 10 µL/min). The final level of the first immobilization was about 10,000 RU.

The three compounds were diluted in PBST 0.1% DMSO at the highest concentration of 10 mM. First, one saturating concentration of each compound (10 µM or 3.3 µM) was injected on the chip surface for 480 s at 25 µL/min. The sensorgrams were baseline corrected by subtracting the signal from an empty reference surface to account for non-specific binding and bulk effect used as reference, and the dissociation constants (Kd) were obtained from the fitting of the sensorgrams with the 1:2 heterogeneous ligands model implemented in the Sierra Analyzer 3.4.5. software. Then, three or more experimental sessions were performed. The three compounds were injected at different concentrations (from 41 nM to 10 µM, at 1:3 serial dilutions) on separate surface spots in parallel. Again, the Sierra Analyzer 3.4.5. software was used to perform a global fitting of the sensorgrams at all the different concentrations to obtain the association and dissociation rate constants (k_a_ and k_d_) and the equilibrium dissociation constant (K_D_). All the experimental procedures used in the sessions and the corresponding raw data were saved by the Sierra SPR-32pro instrument and are available.

### 4.3. Crystallization and Structure Determination of hTPSB2 (Apo and Ligand-Bound Forms)

To investigate the structural basis of tryptase inhibition, we determined X-ray crystal structures of human tryptase beta-2 (hTPSB2) in its apo form and in complex with compound #**1**. These structures were used both as reference models for docking simulations and to provide experimental validation of the predicted binding modes.

Crystallization trials were conducted using a standard screen of approximately 1200 conditions and literature-based refinements. Initial conditions were optimized by systematically varying temperature, protein concentration, drop ratio, pH, and precipitant concentrations to improve crystal quality.

Crystals of apo hTPSB2 and hTPSB2-compound #**1** complex were flash-vitrified and measured at 95 K following PROTEROS Standard Protocols. X-ray diffraction data were collected at the SWISS LIGHT SOURCE (SLS, Villigen, Switzerland) under cryogenic conditions. The crystals belonged to space group P 31, and data were processed using autoPROC, XDS, and AIMLESS (Table 2).

The phase information necessary to determine and analyze the structure was obtained by molecular replacement. A published structure of hTPSB2 was used as a search model. Subsequent model building and refinement was performed according to standard protocols with COOT and the software package CCP4-8, respectively. For the calculation of the free R-factor, a measure to cross-validate the correctness of the final model was used; about 0.5% of measured reflections were excluded from the refinement procedure. Automatically generated local NCS restraints have been applied (keyword “ncsr local” of newer REFMAC5 versions). The water model was built with the “Find waters” algorithm of COOT by putting water molecules in peaks of the Fo-Fc map contoured at 3.0 followed by refinement with REFMAC5 and checking all waters with the validation tool of COOT. The criteria for the list of suspicious waters were B-factor greater 80 Å 2, 2Fo-Fc map less than 1.2 σ, distance to closest contact less than 2.3 Å or more than 3.5 Å. The suspicious water molecules and those in the ligand-binding site (distance to ligand less than 10 Å) were checked manually. The Ramachandran plot of the final model calculated with Molprobity shows 97.21% of all residues in the favored region and 2.79% in the allowed region. There are no outliers in the Ramachandran plot.

The atomic models have been deposited at the Protein Data Bank (PDB) and are available under the accession number 9QFV for the apo structure and 9QFU for the complex with compound #**1**.

### 4.4. Docking Studies

Molecular docking studies were performed to predict the binding modes of compound #**1**, compound #**2m**, and compound #**2d** using the X-ray crystal structure of human tryptase beta-2 (hTPSB2) in its apo form. Compounds were converted to 3D structures and prepared by using Schrödinger’s LigPrep tool. This process generated multiple states for stereoisomers, tautomers, ring conformations (one stable ring conformer by default), and protonation states. In particular, another Schrödinger package, Epik 6.8, was used to assign tautomers and protonation states that would be dominant at a selected pH range (pH = 7 ± 1). Ambiguous chiral centers were enumerated, allowing a maximum of 32 isomers to be produced from each input structure. Then, energy minimization was performed with the OPLS3 forcefield.

The protein was prepared using Maestro Protein Preparation Wizard. Hydrogen atoms were added, and water molecules were removed from the protein structure.

The GENEOnet 1.2 tool was employed to define protein-binding pockets and guide the docking experiments [16]. Developed by Dompé Farmaceutici SpA, this proprietary software integrates the geometric and explainability features of Group Equivariant Non-Expansive Operators (GENEOs) within a network architecture, creating a novel, knowledge-driven machine learning paradigm. By leveraging critical chemical–physical properties such as lipophilicity, hydrophilicity, and electrostatics, GENEOnet effectively identifies and prioritizes binding sites. For each parameter, a distinct GENEO [17] is employed to pinpoint regions with optimal values, enhancing the precision of pocket detection.

The docking simulations were performed by using LiGen. LiGen, a proprietary software developed by Dompé, implements a geometrical fitting procedure combined with a rigid body minimization. The Chemical Score, representing the ligand-binding interaction energy, is calculated using an in-house-developed scoring function after an initial rigid-body minimization is performed to optimize the docked ligand within the binding site. All poses that do not fulfill geometric fitting or threshold values of user-defined specific parameters are discarded.

Covalent docking was performed using the Covalent Docking 10.3 module of Schrodinger.

## 5. Conclusions

We have developed a novel and reliable SPR-based binding assay to evaluate potential ligands to human tryptase, measuring the underlying binding constants and stoichiometry of the ligand–protein complex. The assay proved to be a valuable tool to integrate in silico predictions and obtain important experimental insights into both the strength and the mode of interaction of ligands with the tetrameric human tryptase that was finally validated by X-ray crystal structures of one tryptase–inhibitor complex. This can provide a surrogate or additional method to investigate and study the binding properties and stoichiometry of small molecules against their target, limiting the expensive costs of structural microscopy techniques.

This integrated and multimodal approach can be used for future screening campaigns to identify new ligands of the human tryptase, levering the synergism between the in silico prediction and the experimental validation.

## Figures and Tables

**Table 1:** Summary of the kinetic constants obtain from the fitting curves in different experimental sessions. The last row for each compound represents the mean ± standard deviation (SD) of the individual measurements.

Compound	k_a_ [1/Ms]	k_d_ [1/s]	K_D_ [nM]	Rmax [RU]
#**1**	1.187 × 10^4^	2.6 × 10^−4^	22.0	48.0
	1.287 × 10^4^	0.8 × 10^−4^	6.5	45.8
	1.143 × 10^4^	1.0 × 10^−4^	8.5	38.6
	1.2 ± 0.08 × 10^4^	1.5 ± 0.99 × 10^−4^	12.3 ± 8.4	44.1 ± 4.9
#**2m**	729.0	7.6 × 10^−5^	105.0	29.3
	721.0	2.1 × 10^−5^	29.1	22.4
	633.1	7.2 × 10^−5^	114.0	27.2
	694 ± 53.2	5.3 ± 3 × 10^−5^	81 ± 46.4	26.3 ± 3.5
#**2d**	2.611 × 10^4^	8.5 × 10^−5^	3.2	27.4
	2.638 × 10^4^	5.0 × 10^−5^	1.9	40.0
	2.931 × 10^4^	4.7 × 10^−5^	1.7	34.1
	2.397 × 10^4^	4.0 × 10^−5^	1.7	29.6
	2.7 ± 0.21 × 10^4^	5.6 ± 2.01 × 10^−5^	2.1 ± 0.7	32.8 ± 5.6

**Table 2:** Data collection and processing statistics for apo hTPSB2 and hTPSB2-compound #**1** complex.

X-Ray Source	PXII/X10SA (SLS ^1^)
Wavelength [Å]	1.0001
Detector	Dectris EIGER2 Si 16M
Temperature [K]	100
Space group	P 31
Cell: a; b; c [Å]	78.55; 78.55; 165.69
γ; β; α [°]	90.0; 90.0; 120.0
Resolution [Å]	1.98 (2.02–1.98)
Unique reflections	79,329 (3995)
Multiplicity	6.6 (6.3)
Completeness [%]	100.0 (100.0)
Rpim [%]	7.7 (93.0)
Rsym [%]	18.3 (214.5)
Rmeas [%]	19.9 (234.0)
CC1/2 [%]	99.50 (53.20)
Mean (I)/sd	7.8 (1.3)

^1^ SWISS LIGHT SOURCE (SLS, Villigen, Switzerland).

## Data Availability

Data are available upon request.

## References

[1] Elieh Ali Komi D., Wöhrl S., Bielory L. (2020). Mast Cell Biology at Molecular Level: A Comprehensive Review. Clin. Rev. Allerg. Immunol..

[2] Atiakshin D., Buchwalow I., Samoilova V., Tiemann M. (2018). Tryptase as a Polyfunctional Component of Mast Cells. Histochem. Cell Biol..

[3] Hallgren J., Pejler G. (2006). Biology of Mast Cell Tryptase. FEBS J..

[4] Levi-Schaffer F., Piliponsky A.M. (2003). Tryptase, a Novel Link between Allergic Inflammation and Fibrosis. Trends Immunol..

[5] Ribatti D. (2024). Tryptase and Tumor Angiogenesis. Front. Oncol..

[6] O’Connell M.P., Lyons J.J. (2022). Resolving the Genetics of Human Tryptases: Implications for Health, Disease, and Clinical Use as a Biomarker. Curr. Opin. Allergy Clin. Immunol..

[7] Cao M., Gao Y. (2024). Mast Cell Stabilizers: From Pathogenic Roles to Targeting Therapies. Front. Immunol..

[8] Giardina S.F., Werner D.S., Pingle M., Bergstrom D.E., Arnold L.D., Barany F. (2018). A Novel, Nonpeptidic, Orally Active Bivalent Inhibitor of Human β-Tryptase. Pharmacology.

[9] Palmer J.T., Rydzewski R.M., Mendonca R.V., Sperandio D., Spencer J.R., Hirschbein B.L., Lohman J., Beltman J., Nguyen M., Liu L. (2006). Design and Synthesis of Selective Keto-1,2,4-Oxadiazole-Based Tryptase Inhibitors. Bioorg Med. Chem. Lett..

[10] Schasfoort R.B.M. (2017). Handbook of Surface Plasmon Resonance.

[11] Homola J., Piliarik M., Homola J. (2006). Surface Plasmon Resonance Based Sensors.

[12] Sofiyev V., Kaur H., Snyder B.A., Hogan P.A., Ptak R.G., Hwang P., Gochin M. (2017). Enhanced Potency of Bivalent Small Molecule Gp41 Inhibitors. Bioorganic Med. Chem..

[13] Guo X.-K., Zhang Y. (2022). CovBinderInPDB: A Structure-Based Covalent Binder Database. J. Chem. Inf. Model..

[14] Costanzo M.J., Yabut S.C., Zhang H.-C., White K.B., De Garavilla L., Wang Y., Minor L.K., Tounge B.A., Barnakov A.N., Lewandowski F. (2008). Potent, Nonpeptide Inhibitors of Human Mast Cell Tryptase. Synthesis and Biological Evaluation of Novel Spirocyclic Piperidine Amide Derivatives. Bioorganic Med. Chem. Lett..

[15] Akçay G., Belmonte M.A., Aquila B., Chuaqui C., Hird A.W., Lamb M.L., Rawlins P.B., Su N., Tentarelli S., Grimster N.P. (2016). Inhibition of Mcl-1 through Covalent Modification of a Noncatalytic Lysine Side Chain. Nat. Chem. Biol..

[16] Bocchi G., Frosini P., Micheletti A., Pedretti A., Gratteri C., Lunghini F., Beccari A.R., Talarico C. (2022). GENEOnet: A New Machine Learning Paradigm Based on Group Equivariant Non-Expansive Operators. An Application to Protein Pocket Detection. arXiv.

[17] Bergomi M.G., Frosini P., Giorgi D., Quercioli N. (2019). Towards a Topological–Geometrical Theory of Group Equivariant Non-Expansive Operators for Data Analysis and Machine Learning. Nat. Mach. Intell..

